# Posttraumatic Stress Disorder, Parenting, and Marital Adjustment among a Civilian Population

**DOI:** 10.3389/fpsyg.2017.01655

**Published:** 2017-10-04

**Authors:** Michal Hershkowitz, Rachel Dekel, Shimon Fridkin, Sara Freedman

**Affiliations:** School of Social Work, Bar Ilan University, Ramat Gan, Israel

**Keywords:** PTSD, marital satisfaction, parenting, civilian trauma

## Abstract

While psychopathology in general is linked to poorer marital and parental satisfaction, there is a paucity of data regarding these interactions in parents with Posttraumatic stress disorder (PTSD). The current study addresses this issue among a civilian population. Two hundred trauma-exposed parents, mean age of 37.2, 62% mothers, were assessed using self-report questionnaires, for background variables, PTSD symptoms using the Posttraumatic Stress Diagnostic Scale (PDS), depression symptoms (Beck Depression Inventory, BDI), marital satisfaction (Dyadic Adjustment Scale, DAS-7), parenting behavior (Alabama Parenting Questionnaire, APQ-9), and parenting satisfaction (Parenting Satisfaction Questionnaire). We hypothesized that positive parenting behavior and parenting satisfaction would be negatively correlated with PTSD symptom levels, and that this relationship would be mediated by marital satisfaction; the independent effects of depression on marital and parenting functioning were also examined. Data was analyzed using structural equation modeling (SEM). Results indicated that PTSD was related to poorer parenting behavior (B = 0.089, *p* = 0.033), depression had a negative impact on parenting satisfaction (B = 0.983, *p* = 0.003), and marital satisfaction (B = −0.672, *p* = 0.004), and marital satisfaction fully mediated the relationship between depression and parenting. The findings demonstrated that the effects of PTSD can cast a pall not only over the individual but over the entire family. Interventions are needed to address these issues.

## Introduction

Posttraumatic stress disorder (PTSD) is broadly associated with difficulties in family functioning. Studies have specifically examined the effects on significant others, showing that spouses of veterans with PTSD also report elevated levels of PTSD, as well as emotional stress and caregiver burden (Ben Arzi et al., 2000; Campbell and Renshaw, 2013). Vietnam veterans with severe PTSD have reported reduced marital and sexual satisfaction (Koenen et al., 2003). Numbing symptoms in particular appear to impact interpersonal relationships, with studies showing that a reduction in numbing symptoms, as a result of therapy, was related to an increase in relationship satisfaction in Vietnam veterans (Lunney and Schnurr, 2007), and that increased numbing symptoms predicted poorer relationship satisfaction in a cohort of National Guard service members and their partners (Campbell and Renshaw, 2013).

In terms of effects on children, studies have shown that children of male veterans suffering from PTSD are more likely to show behavior problems (Davidson and Mellor, 2001; Lambert et al., 2014), higher anxiety (Ahmadzadeh and Malekian, 2004; Lambert et al., 2014), and greater aggression (Beckham et al., 1997; Ahmadzadeh and Malekian, 2004) than children whose male veteran fathers do not suffer from PTSD.

In the few studies examining parenting in individuals with PTSD, two have found that numbing symptoms were related to poorer reported relationship quality and parenting satisfaction in male veterans (Samper et al., 2004; Taft et al., 2008). Other studies of parenting in veterans have shown that lower parental satisfaction was related to hyperarousal symptoms in female veterans (Berz et al., 2008). In a prospective study of Army National Guard fathers, increases in PTSD symptoms were associated with poorer couple adjustment and greater perceived parenting challenges over time (Gewirtz et al., 2010).

Only two studies, to our knowledge, have examined parenting and PTSD among civilian populations. In a study of a large civilian population exposed to trauma, numbing was not found to be predictive of relationship quality or parent–child conflict, after controlling for work-related stress and finances (Lauterbach et al., 2007). In a study of abused women, their exposure to violence during childhood was related to reduced parental satisfaction (Waldman-Levi et al., 2013).

Although studies have shown that in the general population marital satisfaction and parenting are closely related (Coln et al., 2013), these relationships have not been examined in PTSD populations, with the exception of one study (Gewirtz et al., 2010), which found that among a military population, increases in PTSD over time were related to poorer couple adjustment and poorer effective parenting. Although couple adjustment and parenting were correlated, couple adjustment did not mediate the relationship between PTSD and parenting. In addition, no study to our knowledge has investigated marital satisfaction, parenting and PTSD among a civilian population. The majority of studies have included veterans, most of whom are men, and therefore the impact of PTSD on civilian women's marital satisfaction and parenting is less well understood.

Viewing these studies collectively, it seems likely that PTSD is directly related to poorer marital and parental satisfaction, as well as to poorer parenting behavior. Although the relationship between marital satisfaction and parenting may be bidirectional, most previous studies of family functioning have examined the effects of marital adjustment on parenting, thus there is an assumption that a poorer relationship between spouses mediates the relationship between psychopathology and parenting (Kwok et al., 2015). However, this finding did not emerge in the only study that has examined this with people suffering from PTSD (Gewirtz et al., 2010). Since psychopathology in general (as opposed to PTSD in particular) is related to poorer marital and parenting adjustment (Papp et al., 2004), it is also important to assess the role of depression. Approximately 50% of people who suffer from PTSD also suffer from a concurrent major depressive disorder, and depression in particular is likely to impact perceived satisfaction from interpersonal relationships (Kessler et al., 2005). No study as far as we are aware has examined the impact of depression as well as PTSD on family functioning.

In this study, we attempted to address these gaps, by examining the relationship between parenting and PTSD among a civilian population. We hypothesized that positive parenting behavior and parenting satisfaction would be related to each other, and both would be negatively correlated with PTSD symptom levels. We further hypothesized, that this relationship would be mediated by marital satisfaction. Since depression is both comorbid with PTSD and negatively impacts family functioning, the independent effects of depression on marital and parenting functioning were also examined.

## Methods

### Participants

Trauma-exposed parents were recruited from mental health clinics throughout Israel or via online trauma or parenting forums; they were then asked to either fill out questionnaires or respond to questions via a secure internet site. The study was carried out in accordance with the recommendations of the Declaration of Helsinki with written informed consent from all subjects; ethical approval was obtained from the IRBs in three institutions that housed the mental health clinics: Hadassah Medical Organization, Jerusalem, Hillel Yafe Hospital, Hadera, and Ichilov Hospital, Tel Aviv, as well as from the Ethics Committee, the School of Social Work, Bar Ilan University for subjects recruited online.

### Sample

Questionnaires were distributed to 330 individuals, of whom 200 responded (60.6%); the sample size was therefore *n* = 200. Out of 200 respondents, 31 (15%) did not provide their gender data. Of those who did, 45 (23%) were men and 124 (62%) were women [$\chi_{\left(1\right)}^{2}$ = 51.5, *p* < 0.001]. Eight participants (4%) did not report their age; the age of the remaining participants ranged from 23 to 59, with a mean of 37.20 (*SD* = 7.32). The number of children in each family ranged between one and nine, with an average of 3.33. One hundred eighty-one (91%) were married [$\chi_{\left(3\right)}^{2}$ = 472.85, *p* < 0.001], and 156 (78%) had a post-high-school education [$\chi_{\left(5\right)}^{2}$ = 422.58, *p* < 0.001]. One hundred and thirty-three (62%) reported at least an average socioeconomic status [$\chi_{\left(3\right)}^{2}$ = 125.54, *p* < 0.001].

### Measures

Demographic questionnaire: A questionnaire constructed for the purposes of this study assessed demographic information such as gender, age, education, marital status, economic status, number, and age of children. Traumatic events were assessed using the Posttraumatic Stress Diagnostic Scale (PDS), which gives a list of potential traumatic events, and includes a category of “other” for events not included in the list. Events reported included illness (*N* = 35, 17.5%), terror attack (*N* = 32, 16%), accidents (*N* = 14, 7%), murder or suicide of close person (*N* = 30, 15%), sexual abuse (*N* = 37, 18.5%), physical abuse (*N* = 12, 6%), and other (*N* = 36, 18%).

The Posttraumatic Stress Diagnostic Scale, PDS (Foa et al., 1993). This scale assesses all 17 symptoms of PTSD (DSM IV), which are rated on a 4-point scale, ranging from 0 (*not at all*) to 3 (*almost always*). Cronbach's alpha for this study was 0.96.

The Beck Depression Inventory—II (BDI, Beck et al., 1993). This 21-item questionnaire assesses symptoms of depression. Cronbach's alpha for this study was 0.94.

The Dyadic Adjustment Scale—Short Form (DAS-7, Hunsley et al., 1995). This abbreviated version of the full questionnaire that measures marital satisfaction. Its seven items assess marital agreement (three items), joint activities (three items), and general happiness with the relationship (one item); all items are scored on a six-point scale. The final score is the average of all seven items. Cronbach's alpha in this study was 0.86.

The Parenting Satisfaction Questionnaire (Kurdek and Fine, 1991). This questionnaire consists of three questions regarding satisfaction derived from fulfilling one's role as a parent. Answers are given on a seven-point scale; Cronbach's alpha for this study was 0.86.

The Alabama Parenting Questionnaire—Short Form Questionnaire (APQ-9, Elgar et al., 2006). This abbreviated version of the full version APQ (Shelton et al., 1996) assesses parenting behavior The questionnaire has nine items, examining three aspects of parenting: positive parenting, lack of consistency in discipline, and lack of supervision. For the purpose of this study, the questionnaire was translated into Hebrew and checked with back translation and two independent, bilingual judges. The questionnaire items were answered on a five-point scale. Cronbach's alpha in this study was 0.69 (positive parenting), 0.60 (inconsistent discipline), and 0.65 (lack of supervision).

### Data analysis

All hypotheses and research questions were treated via structural equation modeling (SEM) using the IBM SPSS Amos 23 for Windows software package. Two main advantages of SEM are extensive assessment of an *a priori* specified model, which is clearly advantageous for the model specified in this study; likewise, SEM corrects for error variance, and thus more accurately identifies parameters of interest.

We used five fit indices: model chi-square, the root mean square error of approximation (RMSEA; Cudeck and Browne, 1992), the normed fit index [NFI, the non-normed fit index (NFI, Bentler and Bonett, 1980; Bollen, 1990) and the comparative fit index (CFI; Bentler, 1990)]. For the RMSEA statistic, lower values indicate a better model fit, when the value 0.08 is the traditional threshold for an acceptable fit (and 0.05 for a close fit). For the NFI and CFI statistics, better-fitting models achieve higher values, with 0.90 and 0.95 as traditional thresholds for an acceptable and a close model fit, respectively (Kline, 2011). Also for NFI statistics, better-fitting models achieve higher values, with 0.90 and 0.95 as traditional thresholds for an acceptable and a close model fit, respectively (Bentler and Bonett, 1980).

In the hypothesized model, PTSD, depression, parenting behavior and satisfaction, and marital satisfaction, were specified as observed variables with multiple indicators.

To control for demographics, we included age and number of children in the analysis, allowing these variables to link both the exogenous and the endogenous variables in the structural model. Gender was not included since the small number of men did not allow statistical analyses.

Bootstrapping procedures have been advocated as an approach that is well suited for testing hypothesized mediating effects (Shrout and Bolger, 2002; Mackinnon et al., 2004; Preacher and Hayes, 2008). Therefore, bootstrapping procedures were employed to test this hypothesis.

## Results

Table 1 presents the correlations between study variables.

**Table 1:** Correlations among study variables (*N* = 200).

**Variable**	**1**	**2**	**3**	**4**	**5**	**6**
1. Age	–					
2. Number of children	0.35^**^	–				
3. Depression	0.14^*^	−0.09	–			
4. PTSD	0.16^*^	−0.15^*^	0.85^**^	–		
5. Marital satisfaction	−0.14^*^	0.08	−0.45^**^	−0.37^**^	–	
6. Parenting behavior	−0.18^*^	−0.17^*^	−0.28^**^	−0.24^**^	0.31^**^	–
7. Parenting satisfaction	0.01	0.00	−0.54^**^	−0.46^**^	0.54^**^	0.35^**^

* p < 0.05;

** *p < 0.01*.

**Figure 1:**
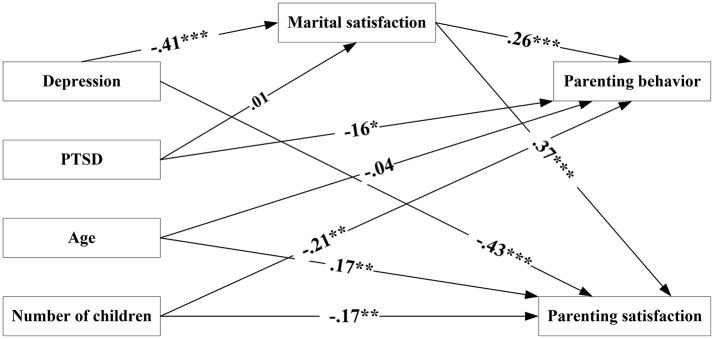
Final structural equation model predicting parenting behavior, parenting satisfaction, and marital satisfaction. *N* = 200, ^*^p < 0.05; ^**^p < 0.01; ^***^p < 0.001.

In light of these correlations, a maximum likelihood estimation was employed to estimate the hypothesized research model. The structural model demonstrated a close model fit, c^2^ = 3.61 (*df* = 4, *N* = 200, *p* = 0.46), CFI = 0.99, NFI = 0.99, RMSEA = 0.00. The final structural model illustrated in Figure 1.

The measures of the final structural equation model fit and the estimated model parameters, including direct and indirect effects, are presented in Table 2.

**Table 2:** Standardized path coefficients for final structural equation model.

**Dependent constructs**	**Independent constructs**				
	**PTSD**	**Depression**	**Age**	**Number of children**	**Marital satisfaction**
**DIRECT EFFECTS**					
Marital satisfaction	0.006	−0.409^**^	–	–	–
Parenting satisfaction	–	−0.433^**^	0.167^*^	−0.171^**^	0.373^**^
Parenting behavior	−0.165^*^	–	−0.040	−0.208^*^	0.257^**^
**INDIRECT EFFECTS**					
Marital satisfaction	–	–	–	–	–
Parenting satisfaction	0.002	−0.153^**^	–	–	–
Parenting behavior	0.001	−0.105^**^	–	–	–

* p < 0.05;

** *p < 0.01*.

PTSD was found to have a negative direct effect on parenting behavior [B = −0.089, SE_B_ = 0.036, bias-corrected bootstrap 95% confidence interval_B_ = (−0.146; −0.004); β = −0.165, SE_β_ = 0.067, bias-corrected bootstrap 95% confidence interval_β_ = (−0.288; −0.009); *p* = 0.033]. Depression was found to have a negative direct effect on marital satisfaction [B = −0.672, SE_B_ = 0.151, bias-corrected bootstrap 95% confidence interval_B_ = (−1.055; −0.420); β = −0.409, SE_β_ = 0.092, bias-corrected bootstrap 95% confidence interval_β_ = (−0.600; −0.253); *p* = 0.004] and parenting satisfaction [B = −0.983, SE_B_ = 0.138, bias-corrected bootstrap 95% confidence interval_B_ = (−1.311; −0.749); β = −0.433, SE_β_ = 0.058, bias-corrected bootstrap 95% confidence interval_β_ = (−0.553; −0.312); *p* = 0.003]. Age was found a positive direct effect on parenting satisfaction [B = −0.028, SE_B_ = 0.009, bias-corrected bootstrap 95% confidence interval_B_ = (0.007; 0.043); β = 0.167, SE_β_ = 0.053, bias-corrected bootstrap 95% confidence interval_β_ = (0.025; 0.250); *p* = 0.029]. Number of children was found a negative direct effect on parenting satisfaction [B = −0.117, SE_B_ = 0.040, bias-corrected bootstrap 95% confidence interval_B_ = (−0.208; −0.047); β = −0.171, SE_β_ = 0.059, bias-corrected bootstrap 95% confidence interval_β_ = (−0.317; −0.076); *p* = 0.005] and parenting behavior [B = −0.050, SE_B_ = 0.015, bias-corrected bootstrap 95% confidence interval_B_ = (−0.082; −0.021); β = −0.208, SE_β_ = 0.061, bias-corrected bootstrap 95% confidence interval_β_ = (−0.312; −0.081); *p* = 0.009]. Marital satisfaction was found to have a positive direct effect on parenting satisfaction [B = 0.516, SE_B_ = 0.080, bias-corrected bootstrap 95% confidence interval_B_ = (0.438; 0.663); β = 0.373, SE_β_ = 0.053, bias-corrected bootstrap 95% confidence interval_β_ = (0.238; 0.465); *p* = 0.007] and parenting behavior [B = 0.126, SE_B_ = 0.036, bias-corrected bootstrap 95% confidence interval_B_ = (0.058; 0.195); β = 0.257, SE_β_ = 0.070, bias-corrected bootstrap 95% confidence interval_β_ = (0.116; 0.403); *p* = 0.009]. The effect of depression on parenting satisfaction [B = −0.347, SE_B_ = 0.097, bias-corrected bootstrap 95% confidence interval_B_ = (−0.612; −0.213); β = −0.153, SE_β_ = 0.042, bias-corrected bootstrap 95% confidence interval_β_ = (−0.273; −0.096); *p* = 0.002] and parenting behavior [B = −0.085, SE_B_ = 0.032, bias-corrected bootstrap 95% confidence interval_B_ = (−0.171; −0.035); β = −0.105, SE_β_ = 0.038, bias-corrected bootstrap 95% confidence interval_β_ = (−0.197; −0.046); *p* = 0.005] was fully mediated by marital satisfaction.

## Discussion

To our knowledge, these results are the first to examine parenting, marital satisfaction and PTSD among a civilian population. The results indicate that, as hypothesized, PTSD is negatively related to parenting behavior, and this finding corroborates previous research conducted among a military population (Gewirtz et al., 2010). It was also hypothesized that PTSD would be related to parenting satisfaction, but this hypothesis was not borne out. In addition, the effect of PTSD on parenting behaviors was direct and not mediated via marital satisfaction. Again, this finding replicates findings from previous research (Gewirtz et al., 2010).

We hypothesized that depression would have a significant impact on family relationships; the results indicate that the impact of depression symptoms is significant not only for couple relationships but also for family relationships in general. Both lowered parenting satisfaction and poorer parenting behavior are related to higher levels of depression; marital satisfaction mediated both of these interactions.

Overall, these results, supporting our hypothesis that trauma-related psychopathology is related to difficulties in family functioning. Higher levels of PTSD are related to more problematic parenting behavior. Poor parenting practices have long-term effects on parent-child relationships and child development (Kaczynski et al., 2006; Gryczkowski et al., 2010), and it is possible that these negative effects in turn impact negatively on PTSD levels.

The impact of depression on family functioning has not been formerly studied among this population. This study showed that satisfaction levels are lowered when depression is higher, a logical finding given that depressed people tend to be less satisfied in general. However, these results also showed that marital satisfaction can buffer the effects of depression on parenting. Depression and PTSD are found to be comorbid in about 50% of PTSD cases, and there are conflicting explanations for this relationship. It is possible that they reflect a common post-trauma psychopathology, or that the depression is secondary to the PTSD. In the current study, depression and PTSD were highly correlated, but appeared to impact family functioning in different ways, thus suggesting two interactive but separate processes.

These results indicate that programs aimed to help marital relationships may have wider implications for overall family functioning as well, and that interventions aimed at reducing PTSD and depression also have the potential to positively affect the patient's family functioning.

This study has several methodological limitations. First, it is a cross-sectional study, and no information is available regarding the timing of the traumatic events in relation to the timing of the participants' marriage and becoming a parent. Secondly, the study participants were not all exposed to the same traumatic event. Thirdly, the participants were recruited from a wide range of backgrounds, and lastly, they were assessed solely via the use of self-report measures. One of these measures, assessing parenting behavior, showed low internal consistency, and this may indicate that the full questionnaire should have been used. These limitations notwithstanding, the novel results which emerged bear replication.

## Author contributions

SaF and MH: study design, implementation, preparation of data file, and writing up of results. RD: study design, and writing up of results. ShF: statistical analyses.

### Conflict of interest statement

The authors declare that the research was conducted in the absence of any commercial or financial relationships that could be construed as a potential conflict of interest.
